# Identification of the *MUC2* Promoter as a Strong Promoter for Intestinal Gene Expression through Generation of Transgenic Quail Expressing GFP in Gut Epithelial Cells

**DOI:** 10.3390/ijms18010196

**Published:** 2017-01-19

**Authors:** Rachel M. Woodfint, Paula R. Chen, Jinsoo Ahn, Yeunsu Suh, Seongsoo Hwang, Sang Suk Lee, Kichoon Lee

**Affiliations:** 1 Department of Animal Sciences, The Ohio State University, Columbus, OH 43210, USA; woodfint.1@osu.edu (R.M.W.); chen.1930@osu.edu (P.R.C.); ahn.134@osu.edu (J.A.); suh.83@osu.edu (Y.S.); 2 Animal Biotechnology Division, National Institute of Animal Science, RDA, Wanju-gun, Jeonbuk 55365, Korea; hwangss@korea.kr; 3 Department of Animal Science and Technology, Sunchon National University, Suncheon 57922, Korea; rumen@sunchon.ac.kr

**Keywords:** mucin 2, intestine-specific, promoter, eGFP, transgenic, Japanese quail

## Abstract

Identification of tissue- and stage-specific gene promoters is valuable for delineating the functional roles of specific genes in genetically engineered animals. Here, through the comparison of gene expression in different tissues by analysis of a microarray database, the intestinal specificity of mucin 2 (*MUC2*) expression was identified in mice and humans, and further confirmed in chickens by RT-PCR (reverse transcription-PCR) analysis. An analysis of *cis*-acting elements in avian *MUC2* gene promoters revealed conservation of binding sites, within a 2.9 kb proximal promoter region, for transcription factors such as caudal type homeobox 2 (CDX2), GATA binding protein 4 (GATA4), hepatocyte nuclear factor 4 α (HNF4A), and transcription factor 4 (TCF4) that are important for maintaining intestinal homeostasis and functional integrity. By generating transgenic quail, we demonstrated that the 2.9 kb chicken *MUC2* promoter could drive green fluorescent protein (GFP) reporter expression exclusively in the small intestine, large intestine, and ceca. Fluorescence image analysis further revealed GFP expression in intestine epithelial cells. The GFP expression was barely detectable in the embryonic intestine, but increased during post-hatch development. The spatiotemporal expression pattern of the reporter gene confirmed that the 2.9 kb *MUC2* promoter could retain the regulatory element to drive expression of target genes in intestinal tissues after hatching. This new transgene expression system, using the *MUC2* promoter, will provide a new method of overexpressing target genes to study gene function in the avian intestine.

## 1. Introduction

The specificity of gene expression for tissue- or cell-types and developmental stages is often regulated by promoters and enhancers. Therefore, identification of tissue- and stage-specific genes and characterization of their promoters is important in order to promote transgene expression in a tissue- and stage-specific manner in genetically engineered animals for the elucidation of transgene function. To identify numerous tissue-specific genes in both mice and humans, comparative microarray database analysis based on the Gene Expression Omnibus (GEO) repository was previously used in our studies, and several novel tissue-specific genes were discovered [1,2]. In addition, our comparative analysis of the promoter region of tissue-specific genes has identified conserved *cis*-acting elements as a major aspect of tissue-specific regulation and expression [3,4,5]. The effectiveness of promoter sequences in directing the expression of genes of interest in specific target tissues or cells was widely proven in transgenic studies that used promoters of adipocyte fatty acid binding protein (*aFABP*, also known as *aP2* and *FABP4*) [6], albumin [6], and insulin [7] genes, to name a few, for targeting adipocytes, liver, and pancreatic beta cells, respectively.

In terms of intestine-specific transgene expression, the regulatory regions of several genes were previously used to direct gene expression. For example, a 12.4 kb promoter-enhancer complex or a 9 kb regulatory region of the mouse *Villin* gene was used to direct expression of transgenes such as reporter genes, the oncogenic *K*-*ras* gene, and the Cre recombinase gene in mice to label the intestine, induce intestinal tumorigenesis, and induce gene knockout within the epithelium [8,9,10,11,12]. The promoter of intestinal fatty acid binding protein (*I-FABP*) has also been used to direct the intestinal expression of transgenes such as growth hormone (*GH*) and cystic fibrosis transmembrane conductance regulator (*CFTR*) genes [13,14]. Although results from studies using the *Villin* promoter indicated high expression of downstream genes in the embryonic stage, which is useful in studying the effects of transgene integration on embryonic development, the entire vertical (crypt-villus axis) and horizontal (duodenum-colon axis) expression requires a relatively large 12.4 kb or 9 kb sequence of the regulatory region. In addition, studies using *I-FABP* promoter reported localization of the transgene mimicking its endogenous localization, but they also reported variability issues regarding the degree of transgene expression and phenotypes. In contrast to the distinct expression patterns of *Villin* and *I-FABP*, mucin 2 (*MUC2*), which is a member of the mucin family encoding gel-forming glycoproteins that aids in the protection of the gastrointestinal tract [15,16], showed a constitutive expression pattern throughout the chicken intestinal tissues in this study. This led us to investigate chicken *MUC2* promoter for a reporter transgene expression in an intestine-specific manner.

Transgenic technology will be a useful method in studying intestinal biology. For example, it will allow for a better understanding of direct and specific in vivo roles of potential genes. This technology can also be used to understand encoding factors involved in intestinal digestion and absorption processes as well as intestinal secretory and endocrine factors. However, intestine-specific promoters for modulating gene expression in the intestinal tissues of avian species have yet to be identified. The objectives of this study were to identify intestine-specific genes in avian species through comparative analysis, generate transgenic Japanese quail that contain the enhanced green florescent protein (*eGFP*) gene under the control of a promoter region of an intestine-specific gene (the 2.9 kb chicken *MUC2* promoter) via a gene transfer using lentiviral particles, and investigate distinct spatiotemporal expression of the *GFP* reporter gene. The identification of promoters that can effectively drive expression of a gene of interest in a space and time-dependent manner will pave the way for the investigation of gene function in the intestine for both agricultural and biomedical purposes.

## 2. Results

### 2.1. Microarray Analysis of Intestine-Specific Mucin Genes in Mouse and Human

Through the analysis of expression of mucin (*MUC*) genes in mouse and human tissues based on the aforementioned microarray GEO DataSets (GDS3142 for mice and GDS596 for humans), *MUC13* and *MUC2* were identified as genes that are predominantly expressed in the small intestine of both mice and humans. The MUC13 mRNA showed 61.2- and 50.3-fold higher expression in the small intestine of mice and humans, respectively. The MUC2 mRNA showed 26.9- and 31.7-fold greater expression in the same tissue (Table 1). As a result, these two mucin genes were selected for further comparative examination of small intestine enrichment among chicken tissues.

### 2.2. Confirmation of Intestine-Specific Expression of Mucin Genes in Chickens

**Figure 1:**
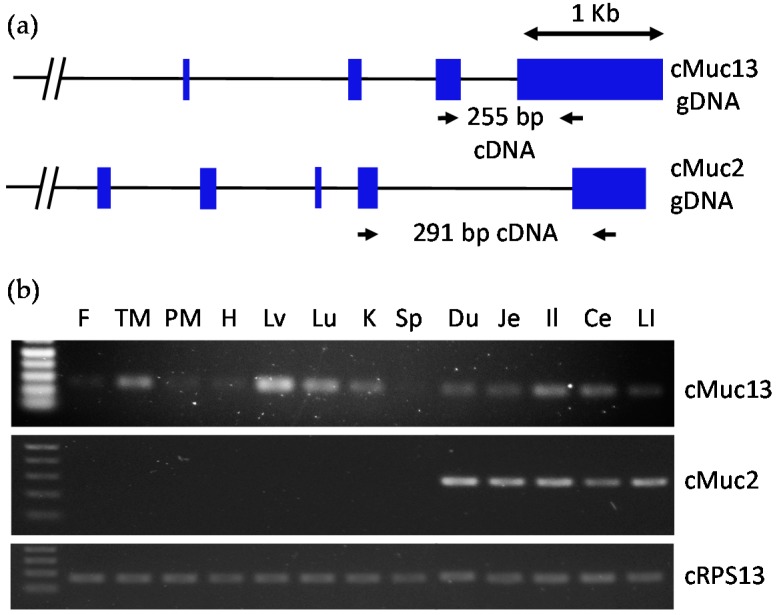
(**a**) Schematic exon-intron diagram of chicken *MUC13* and *MUC2* genes. Locations of inter-exon primers for RT-PCR are marked with arrows and expected sizes of amplified PCR products are indicated. Blue boxes represent exons; (**b**) RT-PCR tissue distribution including fat (F), thigh muscle (TM), pectoralis muscle (PM), heart (H), liver (Lv), lung (Lu), kidney (K), spleen (Sp), duodenum (Du), jejunum (Je), ileum (Il), cecum (Ce), and large intestine (LI). *MUC13* was expressed in numerous tissues outside of the intestinal tissue. *MUC2* was indicated to be intestine-specific, and chicken ribosomal protein s13 (cRPS13) was used as a control.

The expressions of *MUC13* and *MUC2* were confirmed in various tissues of chicken using reverse transcription-PCR (RT-PCR) with primers in exons (Figure 1). The expression of chicken *MUC13* was detected in various parts of the small intestine; however, *MUC13* was also expressed in the thymus, muscle, liver, lung, and kidney of the chicken. In contrast, *MUC2* was exclusively expressed in different parts of the small intestine, ceca, and large intestine. This suggests that *MUC2* is an intestine-specific gene across species, including the avian species, whereas *MUC13* is intestine-specific in mammals, such as mice and humans. Therefore, our comparative analysis using GEO DataSets and RT-PCR has led to the discovery of *MUC2* as a common intestine-specific gene, whose promoter may regulate intestine-specific expression.

### 2.3. Analysis of the MUC2 Promoter

**Figure 2:**
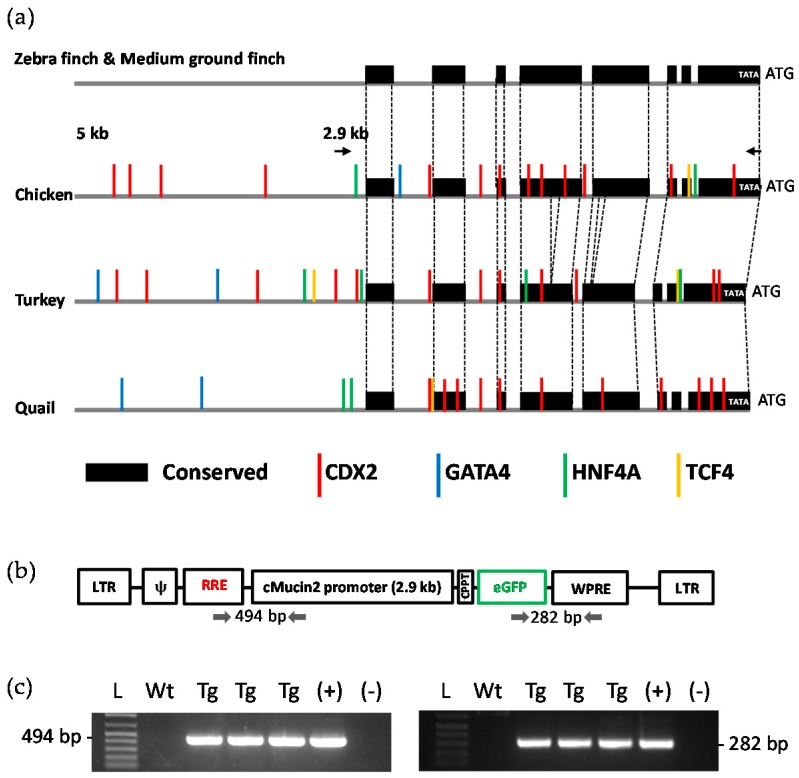
(**a**) Prediction of the distribution of binding sites for an intestine-specific transcription factor. The binding sites of intestine-specific transcription factors (e.g., CDX2 (red), GATA4 (blue), HNF4A (green), and TCF4 (yellow)) are highlighted on the promoter region of the *MUC2* gene from chicken, turkey and quail. Highly conserved DNA sequences between avian species are marked with a black box; (**b**) Viral vector construct. Construct contains 2.9 kb chicken *MUC2* promoter. Located downstream of the promoter is eGFP; (**c**) Confirmation of two primer sets. Two primer sets were utilized to confirm the presence of the transgene in animals. L (DNA ladder), Wt (wild-type), Tg (transgenic quail), + (positive control), − (negative control).

To investigate regulatory regions in the promoter sequence of *MUC2*, the 5 kb upstream regions in birds were compared (Figure 2a). Using the BLAST (Basic Local Alignment Search Tool)-like Alignment Tool (BLAT) search (available online: http://genome.ucsc.edu), highly conserved regions across bird species were identified. As shown by the black boxes in Figure 2a, chicken, turkey, and quail shared multiple regions with other birds, such as the zebra finch and medium ground finch, within the 2.9 kb region that appear to be evolutionarily important. Moreover, predicted binding sites for intestine-specific transcription factors such as caudal type homeobox 2 (CDX2), GATA binding protein 4 (GATA4), hepatocyte nuclear factor 4 α (HNF4A), and transcription factor 4 (TCF4) were significantly distributed in the 2.9 kb sequence, especially in the case of the quail. Thus, this 2.9 kb *MUC2* promoter was chosen for the generation of transgenic quail with intestine-specific expression of enhanced green fluorescent protein (eGFP).

### 2.4. Generation of Transgenic Birds

Genotyping PCR was used to positively identify transgenic birds. The offspring of chimeric founders were screened in order to identify Generation 1 (G1). Of the 105 injected eggs seventeen chimeric founders hatched, a success rate of 16.2%. A single chimeric founder produced one offspring that was positively identified as transgenic. The identification of this animal indicated that the vector construct was able to successfully integrate into at least one chimeric founder. Genotyping PCR was also used to identify transgenic Generation 2 (G2) birds, the progeny of the transgenic G1 bird. G2 birds were hatched normally in a Mendelian ratio (19 wild type and 20 transgenic birds (51.3%), indicating one integration site), with transgenic offspring expressing the eGFP in their intestinal tissue.

### 2.5. Western Blot Analysis of Transgenic Birds for Tissue Distribution

**Figure 3:**
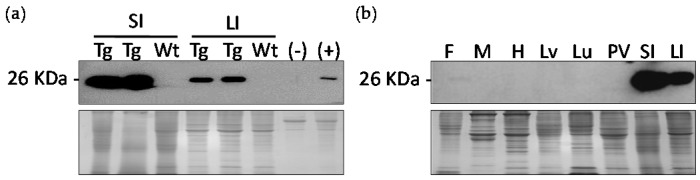
(**a**) Western blot analysis of transgenic Japanese quail intestines compared to wild-type quail intestines. At 26 kDa, eGFP was expressed in the small and large intestine of transgenic quail but not in the wild-type. Coomassie blue staining was utilized to ensure an equal amount of protein was loaded for each sample; (**b**) Western blot analysis of transgenic quail tissue distribution including fat (F), muscle (M), heart (H), liver (Lv), lung (Lu), proventriculus (PV), small intestine (SI), and large intestine (LI). Coomassie blue staining was used to ensure an equal amount of protein was loaded for each sample.

Western blot analysis revealed that the eGFP protein was specific to the intestinal tissues of the transgenic birds. The small intestine and large intestine of day 42 (D42) birds, one wild type and two transgenic birds from the G2 generation were used to perform Western blot (Figure 3a). The results indicated that the eGFP protein was present in the transgenic birds, but not in the wild-type birds.

Western blot was performed using a tissue distribution from a transgenic bird to further determine the expression pattern of eGFP (Figure 3b). The tissues included fat, skeletal muscle, heart, liver, lung, proventriculus, small intestine, and large intestine. The target eGFP protein was detected within the small intestine and large intestine. Therefore, the 2.9 kb *MUC2* promoter was able to successfully direct expression of *eGFP* in only intestinal tissues.

### 2.6. Protein and Fluorescence Detection of eGFP in Epithelial Layer of Intestinal Tissues

**Figure 4:**
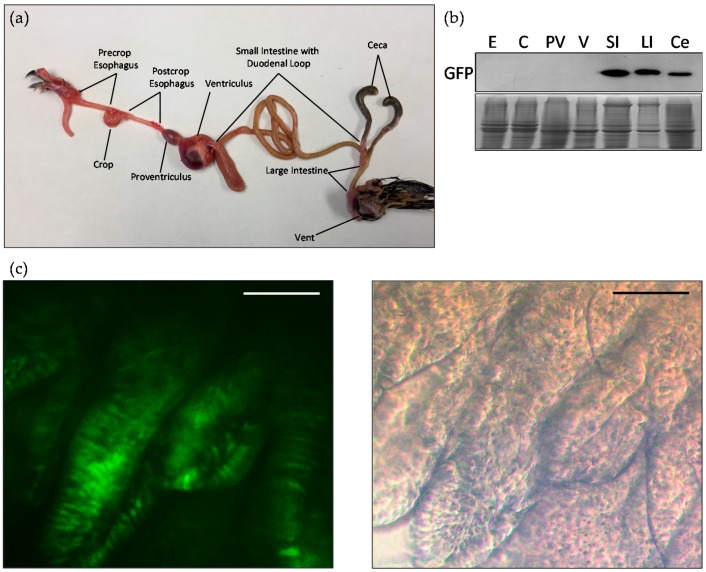
(**a**) Intestinal tract of Japanese quail; (**b**) Western blot analysis of transgenic quail intestinal tissue distribution including esophagus (E), crop (C), proventriculus (PV), ventriculus (V), small intestine (SI), large intestine (LI) and ceca (Ce). Coomassie blue staining was used to ensure an equal amount of protein was loaded for each sample; (**c**) Representative fluorescence and bright-field microscopic images of villi in the transgenic quail small intestine. The scale bar represents 100 µm.

Western blot analysis was also performed to confirm the intestinal specificity of MUC2 protein (Figure 4b). Gastrointestinal tissues, esophagus, crop, proventriculus, ventriculus, small intestine, ceca, and large intestine were collected from a D21 transgenic G2 bird (Figure 4a). Bands appeared at the expected size of 25 kDa for the small intestine, ceca, and large intestine. Coomassie blue staining was used to ensure the same amount of protein was loaded for each sample. Scrapings parallel to the length of the tissue were taken from the intestine of D42 transgenic G2 birds. Under a fluorescent microscope, villi were visible in the intestine of the transgenic animals. The epithelial cells in these villi expressed the eGFP protein (Figure 4c).

### 2.7. Time Point Expression of eGFP

**Figure 5:**
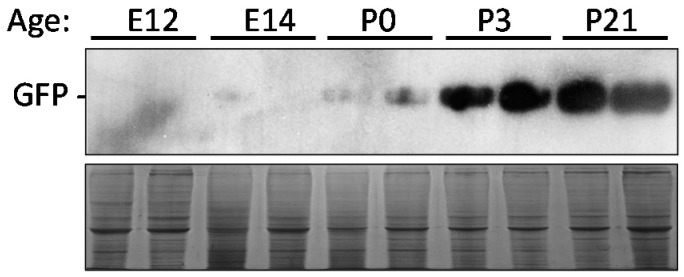
Western blot analysis of different developmental time points of transgenic Japanese quail. These time points include embryonic day (E) 13, E16, post-hatch day (D) 0, D3, and D21. The eGFP expression increases with age. Coomassie blue staining was used to ensure an equal amount of protein was loaded for each sample.

Embryonic day 13 (E13), E16, D0, D3, and D21 transgenic G2 offspring were collected to determine variation within the eGFP expression. Western blot analysis was used to detect any differences. The Western blot indicated that there is an increase in eGFP expression from the embryo to post-hatch D21 (Figure 5).

## 3. Discussion

In this study, the intestinal specificity of mucin 2 (*MUC2*) in avian species was confirmed through microarray data and RT-PCR analysis. Consequently, the 2.9 kb promoter region of chicken *MUC2* was used to generate transgenic quail with an intestine-specific reporter gene expression. Among several mucin genes, intestine-specific expression of the *MUC2* gene was confirmed through our comparative analysis. Also, the avian stomach, or ventriculus, did not express MUC2 mRNA. These findings concur with previous works indicating that MUC2 is expressed on the mucosal surface in both the large and small intestine of humans and mice, but absent or barely detectable in other gastrointestinal tissues, including the stomach [17,18]. Due to the tissue-specific expression of *MUC2*, it was hypothesized that the regulatory promoter region of *MUC2* gene could drive intestine-specific expression of a transgene.

The specific pattern of gene expression in certain types of cells, particular developmental stages, and nutritional conditions has been shown to be regulated by conserved regulatory elements, including a promoter region found in previous studies along with our reports [4,5,19,20,21]. In the current study, conserved promoter regions were identified in the chicken *MUC2* gene that are specifically expressed in intestinal tissues of 7-week-old broiler chickens. Importantly, conservation of *cis*-regulatory regions in *MUC2*, which include binding sites for major transcription factors for intestinal homeostasis and functional integrity such as caudal type homeobox 2 (CDX2), GATA binding protein 4 (GATA4), hepatocyte nuclear factor 4 α (HNF4A), and transcription factor 4 (TCF4), were revealed. The homeodomain protein CDX2 is thought to be an intestine-specific master transcription factor because CDX2 is expressed in the hindgut, and its expression is critical to sustain expression of downstream intestinal transcription factors such as HNF4A. CDX2 deficiency leads to severe hindgut abnormalities and colon dysgenesis [22] and reduced chromatin access that is required for transcription [23]. As a result, binding of other transcription factors including GATA4 and HNF4A is disrupted [24]. Another study reported that CDX2 directs co-occupancy of *cis*-regulatory regions with TCF4 which is essential for intestinal-specific gene expression [25]. In this regard, the regulatory region of *MUC2*, a heavily glycosylated, gel-forming mucin, was selected as the candidate promoter due to high intestine expression indicated in the gene expression profile and conservation of the regulatory region among several avian species. As a result, the chicken *MUC2* promoter containing nine CDX2 (the intestinal master transcription factor; [23]), two HNF4A, one GATA4, and one TCF4B binding sites could successfully promote expression of GFP in the large and small intestine of quail.

Japanese quail, *Coturnix c. japonica*, were used in this study because they reach sexual maturity quickly, are small in size, have a short incubation period, and have a high egg laying capacity. Also, the quail intestine appears to be permissive for the chicken promoter due to a high sequence homology between chicken and quail *MUC2* promoters. Our lab has been able to successfully generate transgenic quail for a number of different studies [4,19,20]. This includes other studies where tissue-specific genes were identified [4]. These studies were attributed to high efficiency of stable integration of recombinant lentiviral particles into the host genome, capacity of infecting both dividing and non-dividing cells, and self-inactivating properties [21,26]. This allowed lentiviral vectors to become reliable and safe gene delivery vehicles for either ubiquitous or tissue-specific expression of transgenes in multiple studies, including our current study [4,19,21,27,28].

The results of this study also indicated that the stimulation of transgene expression through the chicken *MUC2* promoter increases with age post-hatch. In a study by Jiang et al. [29], it was indicated that endogenous chicken MUC2 mRNA expressions increased throughout embryonic development and continued to increase post-hatch. It was noted that following post-hatch day 7, the MUC2 expression remained high. Similarly in this study, post-hatch day 3 and day 21 both exhibited high expression of eGFP protein which was regulated by the chicken *MUC2* promoter. Thus, these results suggest that the *MUC2* promoter can be used to promote target gene expression in the intestines at post-hatch ages.

In summary, by generating transgenic quail, we have demonstrated that the promoter of chicken *MUC2* contains regulatory elements that direct expression to the small intestine, ceca, and large intestine of quail in a developmental stage-dependent manner. These results show that the basic mechanisms that mediate intestine-specific expression are conserved between avian species. The relatively short promoter of the chicken *MUC2* isolated in this study offers a powerful tool for labeling intestinal cells and targeting expression in the intestines of quail as well as other avian species. In this regard, the epithelial specificity of *MUC2* could be a valuable mechanism used to drive the expression of other advantageous molecules. This could provide beneficial information for the poultry industry and potentially improve production by modulating expression of genes that increase food intake, digestibility, gut mobility, gut development, and nutrient uptake. Regarding increasing food safety issues related to pathogenic bacteria in poultry, genes encoding innate anti-bacterial peptides can be delivered in vivo through this current expression system.

## 4. Materials and Methods

### 4.1. Animal Use and Ethics Statement

Animal care and use procedures were approved by the Institutional Animal Care and Use Committee (IACUC) at The Ohio State University (Protocol: 2015A00000135, 12 January 2016, IACUC). Japanese quail (*Coturnix coturnix japonica*) were housed at The Ohio State University Poultry Facility in Columbus, Ohio. A standard starter or breeder diet and water was provided to the animals *ad libitum*. Sacrificed animals were euthanized via CO_2_ inhalation followed by cervical dislocation for tissue collection.

### 4.2. Data Mining Using GEO DataSets

Microarray database in the Gene Expression Omnibus (GEO), a public genomics data repository, was examined to determine intestine-specific genes among mucin (*MUC*) genes, as described in our previous reports [1,2]. In particular, GEO DataSet (GDS) 3142 for the mouse *MUC* genes and GDS3113 for the human *MUC* genes were obtained from the NCBI website, and gene expression profiles for eight tissues (small intestine, spleen, muscle, liver, brain, lung, kidney, and heart) were sorted out based on the normalized expression value. The fold change assigned to gene expression in the small intestine (S. intestine) was estimated by dividing the *MUC* expression value in the small intestine by an average value of the other tissues (Table 1).

### 4.3. Analysis of Transcription Factor Binding Sites on the Promoter Region

The avian DNA sequences in the promoter region of *MUC2*, which include 5 kb upstream region from the start codon (ATG) of *MUC2*, were obtained from the NCBI website (available online: http://www.ncbi.nlm.nih.gov/gene). With these sequences, transcription factor binding sites were predicted using the MatInspector software (Genomatix Software GmbH, Munich, Germany). Among various transcription factors, previously reported intestine-specific transcription factors (CDX2, GATA4, HNF4A, and TCF4) were selected for their binding sites on the *MUC2* promoter sequence [23,24,25].

### 4.4. Total RNA Extraction, cDNA Synthesis, and PCR

To determine tissue specificity of *MUC2*, fat, thigh muscle, pectoralis muscle, heart, liver, lung, kidney, spleen, duodenum, jejunum, ileum, ceca, and large intestine were collected from 7-week-old broiler chickens (*n* = 4). Total RNA from the broiler chicken tissues was isolated using Trizol reagent (Life Technologies, Grand Island, NY, USA) according to the manufacturer’s protocol. RNA quality was assessed by gel electrophoresis, and quantity was measured using a NanoDrop spectrophotometer (NanoDrop Technologies, Wilmington, DE, USA). cDNA was generated by using 1 μg of total RNA and Moloney murine leukemia virus (M-MLV) reverse transcriptase (Invitrogen, Carlsbad, CA, USA) with conditions of 65 °C for 5 min, 37 °C for 52 min, and 70 °C for 15 min. cDNA samples were used to conduct PCR for expression patterns of *MUC2* using the following primers; MUC2-F, 5′-TGACTGAATGTGAAGGAACATGTG-3′ and MUC2-R, 5′-TTCATTTTGATGTTAAGCTGATGG-3′. The amplification conditions for *MUC2* were 95 °C for 1 min, 32 cycles of 95 °C for 25 s, 58 °C for 45 s, and 72 °C for 45 s, and a final extension time of 5 min at 72 °C. As a loading control, ribosomal protein s13 (*RPS13*) was amplified from each tissue using the primers; RPS13-F, 5′-AAGAAGGCTGTTGCTGTTCG-3′ and RPS13-R, 5′-GGCAGAAGCTGTCGATGATT-3′, with amplification conditions of 94 °C for 1 min, 27 cycles of 94 °C for 30 s, 57 °C for 30 s, and 72 °C for 20 s, and a final extension time of 5 min at 72 °C.

### 4.5. Vector Construction and Production of Lentiviral Particles

The 2.9 kb sequence of chicken mucin 2 (*c-MUC2*) gene was amplified from chicken genomic DNA by PCR with a forward primer containing the ClaI site (underlined), 5′-AATCGATTTTAGCAGCAGAG AATCCCCA-3′, and a reverse primer containing the PacI site (underlined), 5′-AGTTAATTAAGGCTAAGG TGGGTGAACTGTGA-3′, and was then cloned into pCR2.1-TOPO vector (Invitrogen). Two restriction enzymes, ClaI and PacI, were used to digest the pCR2.1 recombinant vector. The 2.9 kb *MUC2* promoter replaced a RSV promoter of the pLTReGW lentiviral vector containing *eGFP* that had been constructed previously [23]. The resulting vector, pLT-cMuc2-eGFP, was designed to express the *eGFP* gene specifically in the intestinal tissues through the direction of the *MUC2* promoter. The vector was first transfected into a human intestinal epithelial cell line (Caco-2 cells), and the expression of eGFP was confirmed in these intestinal cells.

Co-precipitation of calcium phosphate and the pLT-cMuc2-eGFP vector was used to produce lentiviral particles. One day prior to the transfection, 293FT cells were plated on 100 mm culture dishes in complete medium. This medium consisted of Dulbecco’s Modified Eagle Medium (DMEM; Life Technologies Inc.) with 10% fetal bovine serum (FBS; Life Technologies Inc.), 1% penicillin/streptomycin (pen/strep; Life Technologies Inc.), 1 mM MEM sodium pyruvate (Life Technologies Inc.), and 0.1 mM MEM non-essential amino acids (Life Technologies Inc.). 9 μg of pLT-cMuc2-eGFP, 9 μg of ViraPower Packaging Mix (Life Technologies Inc.), and 87 μL of 2 M calcium solution (Clontech Laboratories Inc., Mountain View, CA, USA) were added to a final volume of 700 μL of sterile H_2_O (Clontech Laboratories Inc.) to prepare the transfection solution. Then, 700 μL of 2× HEPES-Buffered Saline (HBS) (Clontech Laboratories Inc.) was added dropwise while slowly vortexing the solution. The transfection solution was incubated for 5 min at room temperature and added dropwise to the complete medium. Following 10 h of transfection, the medium was replaced with 5 mL of fresh complete medium. After 48 h, the supernatant was collected and filtered through a 0.22 µm pore-sized filter. The titer of lentiviral supernatant was measured by a standard ELISA method using the Lenti-X p24 Rapid Titer Kit (Clontech Laboratories Inc.) after the non-concentrated viral supernatants were serially diluted. The supernatant was pelleted via centrifugation at 25,000 rpm for 2 h with an ultracentrifuge (L7-65R, Beckman Coulter, Fullerton, CA, USA), resuspended in Opti-MEM as a 100× concentrated lentiviral particle soup, and stored as 40 μL aliquots at −80 °C until further use.

### 4.6. Production of the Founder Quail

Wild-type Japanese quail eggs were cleaned with 70% ethanol and placed laterally on a tray for approximately 4 h at room temperature. Fine-tipped tweezers were used to create a small window of about 4 mm in diameter on the lateral apex of the egg. Then, 2 to 3 µL of the concentrated lentivirus was injected into the subgerminal cavity of 105 stage X embryos using a microinjection system (Tritech Research, Inc., Los Angeles, CA, USA) under a stereomicroscope (Olympus America Inc., Center Valley, PA, USA). The window was sealed with paraffin film, and the eggs were incubated for 14 days at 37.5 °C with 60% relative humidity before being placed in a hatching tray. Seventeen hatched founder chicks were grown to sexual maturity.

### 4.7. Mating and Selection of Transgenic Offspring

The mature founders were mated with one wild-type quail of the opposite sex. Eggs were collected each week and stored in a cooler (13 °C) until incubating. Hatchlings were tagged and reared for 14 days to collect feather pulp for genomic DNA extraction. The pulp from one feather was incubated in 300 μL of cell lysis solution (200 mM NaCl, 50 mM Tris-Cl, 10 mM EDTA, 1% SDS, pH 8.0) containing Proteinase K (0.1 mg/mL, Invitrogen) at 55 °C for at least 2 h. Then, 300 μL of Phenol:Chloroform:Isoamyl Alcohol (25:24:1, *v*/*v/v*) was added to the tube, mixed, and centrifuged at 13,000× *g* for 10 min to remove the protein. The supernatant was transferred to a new tube, and genomic DNA was precipitated by adding 300 μL of isopropanol, inverting, and centrifuging at 13,000× *g* for 5 min. The pellet was washed with 70% ethanol and centrifuged at 13,000× *g* for 2 min. After drying, TE buffer containing RNase A (10 mg/mL, Qiagen, Valencia, CA, USA) was added to dissolve the pellet. The genomic DNA was utilized for genotyping PCR with the primer set, RRE-F, 5′-AATCGCAAAACCAGCAAGAAA-3′ as the forward primer and MUC2-R, 5′-TGTCAAGCAATTTACAGTGAAATATG-3′ as the reverse primer to amplify a 494 bp fragment. Positive offspring were reconfirmed with the primer set, eGFP-F, 5′-GCATGGACGAGCTGTACAAGTA-3′ as the forward primer and WPRE-R, 5′-AATCCTGGTTGCTGTCTCTTTATG-3′ as the reverse primer to amplify a 282 bp fragment. Offspring of the positive G1 progeny from the founders were used as parents to produce transgenic quail for the functional study.

### 4.8. Tissue Collection and Microscopic Examination of GFP Expression

At 3 weeks post-hatch, two wild-type and two transgenic quail were collected to determine expression of eGFP. Fat, skeletal muscle, heart, liver, lung, proventriculus, small intestine, and large intestine were collected from each bird. From the two transgenic birds, the esophagus, crop, ventriculus, and ceca were also collected to examine eGFP expression along the entire gastrointestinal tract. All tissue samples were snap frozen in liquid nitrogen and stored at −80 °C until further analysis. Small intestine samples were collected from transgenic embryos or quail at day 13 of incubation (E13), day 16 of incubation (E16), day 0 post-hatch, day 3 post-hatch (D3), and day 21 post-hatch (D21). Part of the small intestine between the duodenum and jejunum was snap frozen in liquid nitrogen and stored at −80 °C. The intestinal mucosa from the jejunum was scraped parallel to the length of the tissue from D42 transgenic quail and mounted on a glass slide to examine eGFP expression and fluorescence using an AXIO-Vert.A1 optical microscope (Carl Zeiss Microscopy, Thornwood, NY, USA) equipped with an AxioCam MRc5 camera (Carl Zeiss Microscopy).

### 4.9. Western Blot Analysis

Frozen tissue samples were homogenized in ice-cold 1× lysis buffer (62.5 mM Tris, pH 6.8, and 5% SDS) with a Tissuemiser (Thermo Fisher Scientific, Waltham, MA, USA) and mixed with 2× Laemmli buffer (62.5 mM Tris, pH 6.8, 1% SDS, 5% 2-mercaptoethanol, 12.5% glycerol, 0.05% bromophenol blue). Gels stained with Coomassie brilliant blue were used to determine protein loading. Separation of proteins was performed on 12% SDS-PAGE using a mini-Protein system (Bio-Rad Laboratories, Hercules, CA, USA). Following SDS-PAGE and transfer to polyvinylidene fluoride (PVDF) membranes, the membranes were blocked in 4% nonfat dry milk dissolved in Tris-buffered saline-Tween (TBST; 20 mM Tris, 150 mM NaCl, pH 7.4, plus 0.1% Tween 20) for 30 min at room temperature. Then, membranes were incubated overnight at 4 °C with an eGFP primary antibody (1:5000 dilution; Clontech, Mountain View, CA, USA). After washing 6 times for 10 min in TBST, the membranes were incubated in horseradish peroxidase-conjugated secondary anti-mouse IgG (1:5000 dilution; Jackson ImmunoResearch Laboratories Inc., West Grove, PA, USA) at room temperature for 1 h. The membranes were washed with TBST 6 times for 10 min each before detection with Amersham ECL plus Western Blotting Detection Reagents (GE Healthcare Biosciences, Pittsburgh, PA, USA). The blots were exposed to Hyperfilm (GE Healthcare Biosciences) to visualize the target proteins.

### 4.10. Statistical Analysis

A comparison of means of gene expression in multiple tissues was conducted by using one-way ANOVA followed by a Fisher’s protected least significant difference test included in SAS 9.4 software (SAS Institute, Inc., Cary, NC, USA).

## 5. Conclusions

In this study, intestine-specific expression of mucin 2 (*MUC2*) in avian species was identified through comparative analysis of microarray data and RT-PCR analysis. Consequently, the 2.9 kb promoter region of chicken *MUC2* was used to generate transgenic quail for an intestine-specific expression of the *GFP* reporter gene. It was found that the promoter of chicken *MUC2* contains regulatory elements that direct expression to the small intestine, ceca, and large intestine of quail in a developmental stage-dependent manner. The relatively short promoter of the chicken *MUC2* isolated in this study offers a powerful tool to drive expression of target genes to study the function of genes in avian intestine.

## Figures and Tables

**Table 1:** Microarray data analysis for *mucin* gene expression patterns in various tissues based on GDS3142 for the mouse and GDS3113 for the human.

Gene	Fold ^a^	S. Intestine	Spleen	Muscle	Liver	Brain	Lung	Kidney	Heart	*p* Value
**Mouse**										
*Muc13*	61.2	8463 ± 448	131 ± 2	145 ± 5	144 ± 7	124 ± 9	130 ± 8	137 ± 3	157 ± 6	<0.0001
*Muc3*	41.5	3752 ± 1093	82 ± 8	91 ± 4	98 ± 1	77 ± 3	82 ± 2	86 ± 3	117 ± 6	<0.0001
*Muc2*	26.9	2570 ± 195	90 ± 3	93 ± 2	100 ± 6	85 ± 1	86 ± 3	95 ± 5	120 ± 6	<0.0001
*Muc4*	5.9	472 ± 40	70 ± 3	69 ± 4	73 ± 5	71 ± 1	139 ± 11	68 ± 1	71 ± 3	<0.0001
*Muc5ac*	1.2	157 ± 9	114 ± 4	130 ± 3	142 ± 5	113 ± 2	117 ± 5	111 ± 3	165 ± 3	<0.0001
*Mucl1*	1.1	77 ± 7	60 ± 3	78 ± 2	66 ± 2	63 ± 1	66 ± 5	66 ± 1	73 ± 4	<0.05
*Muc5b*	1.1	158 ± 3	147 ± 2	119 ± 3	153 ± 13	104 ± 2	243 ± 21	132 ± 1	141 ± 7	>0.05
*Muc16*	1.0	95 ± 2	74 ± 6	77 ± 1	96 ± 6	73 ± 4	124 ± 7	80 ± 2	114 ± 2	>0.05
*Muc20*	1.0	109 ± 6	108 ± 6	106 ± 7	107 ± 7	105 ± 7	95 ± 3	104 ± 8	125 ± 4	>0.05
*Muc15*	1.0	71 ± 5	63 ± 1	69 ± 3	75 ± 6	71 ± 3	67 ± 2	68 ± 2	76 ± 3	>0.05
*Muc1*	0.6	144 ± 4	98 ± 6	102 ± 7	102 ± 4	85 ± 2	916 ± 33	198 ± 23	127 ± 4	NA
**Human**										
*Muc13*	50.3	100,638 ± 785	204 ± 10	250 ± 12	663 ± 172	277 ± 38	927 ± 659	11,295 ± 945	402 ± 128	<0.0001
*Muc2*	31.7	26,029 ± 501	339 ± 35	1168 ± 318	1498 ± 852	891 ± 324	694 ± 186	613 ± 24	550 ± 45	<0.0001
*Muc20*	2.0	50,305 ± 651	7068 ± 83	9927 ± 289	24,134 ± 413	2257 ± 260	27,753 ± 1550	103,500 ± 1705	5216 ± 354	<0.0001
*Muc7*	0.8	463 ± 78	400 ± 106	687 ± 163	1219 ± 740	494 ± 213	443 ± 143	370 ± 114	484 ± 115	NA
*Muc21*	0.5	429 ± 113	559 ± 320	431 ± 5	1744 ± 315	1048 ± 303	598 ± 14	999 ± 208	450 ± 118	NA
*Mucl1*	0.5	345 ± 96	1267 ± 780	613 ± 87	1032 ± 237	633 ± 83	549 ± 327	415 ± 116	511 ± 142	NA
*Muc4*	0.4	1310 ± 339	470 ± 79	730 ± 372	358 ± 79	314 ± 59	18,487 ± 1343	496 ± 279	357 ± 110	NA
*Muc15*	0.1	374 ± 130	929 ± 152	820 ± 224	540 ± 125	598 ± 121	4075 ± 536	19709 ± 895	611 ± 156	NA

^a^ The fold was calculated by dividing the value of the small intestine (SI) by an average value of the other tissues. NA: not available.

## Abbreviations

*MUC2*	Mucin 2
GFP	Green fluorescence protein
CDX2	Caudal type homeobox 2
GATA4	GATA binding protein 4
HNF4A	Hepatocyte nuclear factor 4 alpha
TCF4	Transcription factor 4
*aFABP*	Adipocyte fatty acid binding protein
*I-FABP*	Intestinal fatty acid binding protein
GEO	Gene Expression Omnibus
GDS	GEO DataSet
